# Supplementary material for: An open-source framework for large-scale, flexible evaluation of biomedical text mining systems
Source: J Biomed Discov Collab. 2008 Jan 29;3:1. doi: 10.1186/1747-5333-3-1 (PMC2276192; doi:10.1186/1747-5333-3-1)
Supplement: Additional file 1 — Evaluation platform source code. The additional file contains the software evaluation framework discussed in this paper. The most current release can be downloaded from SourceForge.net [1]. See the accompanying README file for instructions on installation and use. Also included in the distribution is a collection of UIMA wrappers for some commonly used BioNLP tools and annotated corpora. [file 1747-5333-3-1-S1.zip › bionlp_uima_repository-1.0.1/docs/api/src-html/edu/uchsc/ccp/util/nlp/annotation/filter/OverlappingAnnotationResolver.html]

```
001    package edu.uchsc.ccp.util.nlp.annotation.filter;
002    
003    import java.util.ArrayList;
004    import java.util.HashMap;
005    import java.util.Iterator;
006    import java.util.List;
007    import java.util.Map;
008    import java.util.Set;
009    
010    import edu.uchsc.ccp.util.nlp.annotation.Span;
011    import edu.uchsc.ccp.util.nlp.annotation.TextAnnotation;
012    
013    /**
014     * This class mirrors the functionality of the class by the same name in the edu.uchsc.ccp.uima package
015     * 
016     * @author williamb
017     * 
018     */
019    public class OverlappingAnnotationResolver {
020    
021        private String annotationTypeToResolve;
022    
023        public OverlappingAnnotationResolver(String annotationTypeToResolve) {
024            this.annotationTypeToResolve = annotationTypeToResolve;
025        }
026    
027        public List<TextAnnotation> processAnnotations(List<TextAnnotation> annotations) {
028    
029            /* This hash maps from the annotation Id to the annotation itself */
030            Map<Integer, TextAnnotation> annotationIDMap = new HashMap<Integer, TextAnnotation>();
031            /*
032             * This hash maps from the annotation ID to a flag indicating whether or not this annotation has been flagged for removal. Annotation removal
033             * occurs at the end of the process to avoid ConcurrentModificationExceptions.
034             */
035            Map<Integer, Boolean> annotationIDRemovedMap = new HashMap<Integer, Boolean>();
036    
037            List<TextAnnotation> annotationsToReturn = new ArrayList<TextAnnotation>();
038    
039            List<TextAnnotation> annotationsToProcess = new ArrayList<TextAnnotation>();
040            int annotationID = 0;
041            for (TextAnnotation ta : annotations) {
042                if (ta.getClassMention().getMentionName().equals(annotationTypeToResolve)) {
043                    ta.setAnnotationID(annotationID);
044                    annotationIDMap.put(annotationID, ta);
045                    annotationIDRemovedMap.put(annotationID++, false);
046                    annotationsToProcess.add(ta);
047                } else {
048                    annotationsToReturn.add(ta);
049                }
050            }
051    
052            List<TextAnnotation> annotationsToRemove = new ArrayList<TextAnnotation>();
053    
054            keepLongestAnnotation(annotationsToProcess, annotationIDMap, annotationIDRemovedMap, annotationsToRemove);
055    
056            /* remove unnecessary annotations */
057            List<Integer> annotationIDsToRemove = new ArrayList<Integer>();
058            for (TextAnnotation taToRemove : annotationsToRemove) {
059                annotationIDsToRemove.add(taToRemove.getAnnotationID());
060            }
061    
062            for (TextAnnotation ta : annotationsToProcess) {
063                if (!annotationIDsToRemove.contains(ta.getAnnotationID())) {
064                    annotationsToReturn.add(ta);
065                }
066            }
067    
068            return annotationsToReturn;
069    
070        }
071    
072        /**
073         * keeps the longest annotation for a group of overlapping annotations
074         * 
075         * @param annotationIDMap
076         * @param annotationIDRemovedMap
077         * @param annotationsToRemove
078         * @param jcas
079         */
080        protected void keepLongestAnnotation(List<TextAnnotation> annotations, Map<Integer, TextAnnotation> annotationIDMap,
081                Map<Integer, Boolean> annotationIDRemovedMap, List<TextAnnotation> annotationsToRemove) {
082            Set<Integer> idSet = annotationIDMap.keySet();
083            for (int id : idSet) {
084                TextAnnotation ta = annotationIDMap.get(id);
085                if (ta != null & !annotationIDRemovedMap.get(ta.getAnnotationID())) {
086                    /*
087                     * assign an arbitrary annotation id so we can keep track of which annotations are identical when comparing overlapping annotations
088                     */
089                    Span span = ta.getAggregateSpan();
090                    int longestAnnotation = ta.getAnnotationSpanEnd() - ta.getAnnotationSpanStart();
091                    List<TextAnnotation> overlappingAnnotations = getOverlappingAnnotations(span, annotations);
092    
093                    /* Cycle through overlapping annotations, and remove if shorter than the original */
094    
095                    for (TextAnnotation overlappingAnnotation : overlappingAnnotations) {
096    
097                        /* make sure these are not the exact same annotation */
098                        if (ta.getAnnotationID() != overlappingAnnotation.getAnnotationID()) {
099                            int overlappingAnnotationLength = overlappingAnnotation.getAnnotationSpanEnd()
100                                    - overlappingAnnotation.getAnnotationSpanStart();
101                            if (overlappingAnnotationLength <= longestAnnotation) {
102                                /* remove this annotation from the CAS */
103                                annotationsToRemove.add(overlappingAnnotation);
104                                annotationIDRemovedMap.remove(overlappingAnnotation.getAnnotationID());
105                                annotationIDRemovedMap.put(overlappingAnnotation.getAnnotationID(), true);
106                            } else {
107                                /* keep this annotaition, and remove the previous longest */
108                                annotationsToRemove.add(ta);
109                                annotationIDRemovedMap.remove(ta.getAnnotationID());
110                                annotationIDRemovedMap.put(ta.getAnnotationID(), true);
111                                longestAnnotation = overlappingAnnotationLength;
112                            }
113                        }
114    
115                    }
116                }
117            }
118        }
119    
120        
121        private List<TextAnnotation> getOverlappingAnnotations(Span span, List<TextAnnotation> annotations) {
122            List<TextAnnotation> overlappingAnnotations = new ArrayList<TextAnnotation>();
123            for (TextAnnotation ta : annotations) {
124                if (ta.getAggregateSpan().overlaps(span)) {
125                    overlappingAnnotations.add(ta);
126                }
127            }
128            
129            return overlappingAnnotations;
130        }
131    }
```
